# Non-Financial Conflicts of Interest in Academic Grant Evaluation: A Qualitative Study of Multiple Stakeholders in France

**DOI:** 10.1371/journal.pone.0035247

**Published:** 2012-04-09

**Authors:** Hendy Abdoul, Christophe Perrey, Florence Tubach, Philippe Amiel, Isabelle Durand-Zaleski, Corinne Alberti

**Affiliations:** 1 AP-HP, Hôpital Robert Debré, Unité d’Épidémiologie Clinique, Paris, France; 2 Université Paris Diderot, Sorbonne Paris Cité, Unité d’Épidémiologie Clinique, Paris, France; 3 INSERM, CIE 5, Paris, France; 4 Institut de Cancérologie Gustave Roussy, Unité de Recherche en Sciences Humaines et Sociales, Villejuif, France; 5 AP-HP, Hôpital Bichat-Claude Bernard, Département d’Épidémiologie, Biostatistiques et Recherche Clinique, Paris, France; 6 AP-HP, Département de la Recherche Clinique et du Développement, Unité de Recherche Clinique en Économie de la Santé, Paris, France; IUMSP, University Hospital Lausanne, Switzerland

## Abstract

**Background:**

Peer review is the most widely used method for evaluating grant applications in clinical research. Criticisms of peer review include lack of equity, suspicion of biases, and conflicts of interest (CoI). CoIs raise questions of fairness, transparency, and trust in grant allocation. Few observational studies have assessed these issues. We report the results of a qualitative study on reviewers’ and applicants’ perceptions and experiences of CoIs in reviews of French academic grant applications.

**Methodology and Principal Findings:**

We designed a qualitative study using semi-structured interviews and direct observation. We asked members of assessment panels, external reviewers, and applicants to participate in semi-structured interviews. Two independent researchers conducted in-depth reviews and line-by-line coding of all transcribed interviews, which were also subjected to Tropes® software text analysis, to detect and qualify themes associated with CoIs. Most participants (73/98) spontaneously reported that non-financial CoIs predominated over financial CoIs. Non-financial CoIs mainly involved rivalry among disciplines, cronyism, and geographic and academic biases. However, none of the participants challenged the validity of peer review. Reviewers who felt they might be affected by CoIs said they reacted in a variety of ways: routine refusal to review, routine attempt to conduct an impartial review, or decision on a case-by-case basis. Multiple means of managing non-financial CoIs were suggested, including increased transparency throughout the review process, with public disclosure of non-financial CoIs, and careful selection of independent reviewers, including foreign experts and methodologists.

**Conclusions:**

Our study underscores the importance of considering non-financial CoIs when reviewing research grant applications, in addition to financial CoIs. Specific measures are needed to prevent a negative impact of non-financial CoIs on the fairness of resource allocation. Whether and how public disclosure of non-financial CoIs should be accomplished remains debatable.

## Introduction

Peer review of grant applications is the most widely used method for evaluating clinical research and has been used in industrialized countries to allocate research resources since 1950 [1]. Unlike peer review of manuscripts submitted for publication, the grant-application peer review process has received little research attention [2], [3]. In a 1998 systematic review, Wessely [4] noted that a single abstract on grant-application peer review was presented at the 1997 International Congress on Peer Review. Today, the situation has not improved: since 2001, only five abstracts about grant-application peer review have been reported at this congress (two in 2001, one in 2005, and two in 2009) [5]. Grant-application peer review is an important step in clinical research that is upstream from scientific publication and therefore influences which data will be added to the fund of scientific knowledge.

Many charges have been made against peer review of grant applications [2], [6]–[15]. Applicants have reported that cronyism and other conflicts of interest (CoIs) bias the peer review process [4]. A CoI has been defined as “a set of circumstances that create a risk that professional judgment or actions regarding a primary interest will be unduly influenced by a secondary interest" [16]. CoIs may be individual, institutional, financial, academic, or personal [17]. They are important because they can impact the fairness of the peer-review process. To our knowledge, no empirical study has specifically investigated CoIs in grant-application peer review, particularly CoIs of a non-financial nature.

The French Ministry of Health funded a research project to assess the grant-application review process in France. As part of this project, we conducted a qualitative study to investigate the perceptions and experience of the various stakeholders in the process. Of the topics investigated, CoIs emerged as a significant concern among internal reviewers, external reviewers, and applicants.

The objective of this report is to describe the perceptions and experience of stakeholders regarding non-financial CoIs potentially affecting the grant-application review process, to describe the management of these non-financial CoIs, and to suggest possible solutions.

## Materials and Methods

### Ethics Statement

This qualitative observational study did not involve patients and written consent was not required. Anonymity and confidentiality of the interviews were guaranteed to all participants. An information sheet on the research objectives and confidentiality of study participation was read to each participant at the beginning of each interview. The participant was then asked to give oral consent and to allow audio recording of the interview. The Institutional Review Board of the Paris North Hospitals, Paris 7 University, AP-HP, approved the study protocol, including the information sheet and oral consent procedure (N° IRB00006477).

### French Grant Application System

The French Health Ministry grant program for hospital-based clinical research (*Programme Hospitalier de Recherche Clinique*, PHRC) is composed of two parts: a national program and seven regional programs. Research applications may be submitted to either the national or the relevant regional program. Both the national and the regional programs involve a review of the applications by external French-speaking peer reviewers (from any country) and by a panel composed of a president and several internal reviewers. We confined our study to the national and Paris regional programs. In 2009, the national program allocated about 40 million € with 392 submissions and 176 funded applications, and the Paris regional program allocated about 4 million € with 92 submissions and 24 funded applications.

For the national program, the president of the panel assigns an internal reviewer to each grant application. Then, each application is reviewed and rated by at least two external reviewers selected by the internal reviewer. Grant applicants do not know the identities of their internal or external reviewers; each external reviewer knows the identities of the applicant and internal reviewer but not of the other external reviewer; only the internal reviewer knows the identity of all four people involved. The internal reviewer writes a report on the grant application based on the assessment of the external reviewers. Then, the president of the panel and all the internal reviewers meet to discuss all grant applications. Based on the scientific quality of each project, funding decisions are made during this meeting.

For the Paris regional program, a board composed of the president, several internal reviewers, and the regional director of research assigns two internal and three external reviewers to each application. Grant applicants do not know who reviews their applications. In addition, internal reviewers are masked to external reviewers and each external reviewer is masked to the other external reviewers and to the internal reviewers. Thus external reviewers report anonymously to the internal reviewers. The applications given the highest ratings by the reviewers are then discussed by a panel composed of all the internal reviewers and the board.

### Selection of Study Participants

In 2009, the national and Paris regional programs had 56 internal reviewers and asked 192 external reviewers to review applications submitted by 487 applicants. Eligibility criteria for participation in our study were as follows:.

- For internal reviewers, having been an active member of either the national or the Paris regional committee in 2008 or 2009;
- For external reviewers, having been asked, and having accepted or refused, to review at least one grant application for the national or Paris regional program in 2009 and having reviewed at least one grant application in the last three years;
- For grant applicants, having submitted at least one grant application to the national or Paris regional program in 2009.

All eligible internal reviewers were asked to participate, whereas external reviewers and grant applicants were selected by stratified randomization in order to obtain a broad spectrum of views. Stratification criteria were medical specialty and academic experience (i.e., junior vs. senior university-hospital physician), geographic location (Paris region versus rest of the country), type of stakeholder and, for applicants, rejection of a previous application. Interviews were conducted until the saturation point was reached, i.e., until additional interviews produced no new information [18]. In this type of study, the saturation point is usually reached after about 20 interviews. Here, the saturation point was reached after 38 interviews of internal reviewers, 27 of external reviewers, and 33 of applicants.

### Observation Sessions

One of us (CP) attended the 2009 national and Paris regional committee meetings (a three-day meeting for French National PHRC and a two-day meeting for Paris Regional PHRC) to observe the interactions and to make notes about the debates. No audio recordings were obtained. The notes provided direct information on the review process, as opposed to the rationalized reconstruction of events provided by the reviewers in post hoc interviews.

### Access to Documents

During the observation sessions, we obtained access to the abstracts of the grant applications that were given to the panel members and discussed in the meetings. For the Paris regional program, we also had access to the reports by the external and internal reviewers.

### Interviews

We designed semi-structured interviews based on key themes identified from an analysis of the medical and sociological literature, French grant-application procedures, and official documents. The final interview guide included open questions on seven topics (Table 1). Each eligible participant was asked by email to participate in a study on the overall PHRC peer-review process. To minimize selection bias, no additional information about the study objective was given before enrolment. If the request for participation received no answer, a reminder was sent every 2 weeks, up to a maximum of three reminders.

**Table 1:** Topics covered in the interviews.

**Career and motivation for being involved in the peer review process**
Employment status, past and current
History of applicant/internal or external reviewer
Reasons for being an applicant/internal or external reviewer
**Experience**
Experience in PHRCs and other French institutions as applicant or as internal or external reviewer
Experience in other grant applications as applicant or as internal or external reviewer
**Management of peer review (for reviewers and internal reviewers)**
Methodology and conception of peer review
**Problems of reviewing and perception of biases**
Perception of biases in the grant-application peer review process
**Strengths and weaknesses of the grant-application peer review process**
Strengths of the PHRC review process and of grant-application review in general
Weaknesses of the PHRC review process and of grant-application review in general and specific question regarding conflicts of interests or other peer review weaknesses (perception and experience)
**Improvement of the process**
Suggestions for improvement
Specific questions about blinded peer review, compensation of reviewers, selection of peer reviewers
**Experience with grant applications (for applicants)**
Experience with personal applications: failures and successes
Experience with other grant applications

Interviews were conducted face-to-face at the participants’ workplace or by telephone (36 [37%] interviews) by two of us (CP, a science sociologist; and HA, an epidemiologist trained in semi-structured interviewing by CP). Neutrality of the interviews was ensured by the fact that neither interviewer was involved in the grant-application review process. The interviews began after the panel meetings, in June 2009, and ended in November 2010. They varied in length from 15 to 90 minutes (median, 31 minutes.

The interviews were audio taped and transcribed verbatim anonymously by an individual who was not otherwise involved in the study. Two interviewees refused to be recorded during the interview, and two recordings were of insufficient quality to allow transcription. The written notes taken during these four interviews allowed us to analyze them nevertheless. Biographical information for each participant was collected at the beginning of each interview.

### Analysis of Interviews

The transcribed interviews were analyzed and coded by CP and HA, who used both case-oriented and variable-oriented methods [19]. Each interview was parsed by theme, and recurring themes were identified inferentially. Similarities and differences in thematic contents yielded variables across the cases. The interviewers and another author (PA, sociologist) discussed the development of the themes and variables and validated the process. In addition, cross-validation of the thematic analysis was undertaken at the same time by HA and CP using the text analysis software Tropes® [20]. The results of the analyses were compared and discussed with all the authors. Patterns in, and differences between, interviews were identified. Three topics about CoIs potentially affecting grant-application peer review were defined: perception of CoIs, experience with CoIs, and management of CoIs. The quotes given in this paper were selected by the authors to represent the range of responses. The results are reported according to the RATS qualitative research review guidelines [1], [21].

## Results

### Characteristics of Participants

Of the 205 individuals who were asked to participate, 79 did not reply, 8 refused (usually because of lack of time), 1 was unavailable for participation, and 117 were included. Of those, 98 were interviewed, including 38 internal reviewers, 27 external reviewers, and 33 grant applicants; none declined participation after receiving oral information on the study. The remaining 19 individuals (2 internal reviewers, 9 external reviewers, and 8 applicants) either canceled or failed to attend the interview appointment. Table 2 shows the participant characteristics. Most participants were male (71%) and worked in the Paris region (66%). Among the 107 non-participants, 7 were internal reviewers, 56 external reviewers, and 44 grant applicants. About half of the non-participants came from the Paris region (52%) and 79 (74%) were. Of the 34 applicants who refused to participate, 26 (77%) had submitted PHRC grant applications that were rejected in 2009.

**Table 2:** Characteristics of participants.

	N	N (%)	Internal reviewers, n (N=38)	External reviewers, n (N=27)	Grant applicants, n (N=33)
**Age (years)**	**98**				
30 – 39		8 (8)	0	1	7
40–49		38 (39)	18	9	11
50–59		33 (34)	10	14	9
60–69		9 (9)	5	2	2
Unknown		10 (10)	5	1	4
**Sex**	**98**				
Male		70 (71)	29	19	22
Female		28 (29)	9	8	11
**Geographic area**	**98**				
Paris area		65 (66)	31	14	20
Other regions		33 (34)	7	13	13
**Specialty**	**98**				
Medicine		37 (38)	14	7	16
Surgery		6 (6)	2	3	1
Methodology		11 (11)	9	1	1
Psychiatry		6 (6)	1	2	3
Obstetrics and gynecology		4 (4)	1	1	2
Biology		21 (22)	8	9	4
Anesthesia		10 (10)	2	3	5
Other		3 (3)	1.	1	1
**Job title**	**98**				
Senior teaching-hospital physician		79 (81)	37	23	19
Junior teaching-hospital physician		3 (3)	0	0	3
Physician not working in a teaching hospital		14 (14)	1	4	9
Other		2 (2)	0	0	2
**Experience with grant application review (years)**	**65**				
0–2		13 (20)	11	2	-
3–5		21 (32)	9	12	-
> 5		19 (29)	6	13	-
Unknown		12 (19)	12	0	-
**First grant application submission**	**33**				
Yes		10 (30)	-	-	10
No		23 (70)	-	-	23
**Funding decision in 2009**	**33**				
Accepted		14 (42)	-	-	14
Refused		19 (58)	-	-	19

### Perception of Conflicts of Interest that Might Affect Grant-application Peer Review

During the interviews, most participants (79/98) spontaneously voiced concerns about non-financial CoIs and listed them ahead of all other biases such as those related to scoring, expertise, or notoriety. Industrial or financial CoIs were rarely mentioned by participants and were often viewed as minor or nonexistent in the PHRC review process: *“Normally, [industrial conflicts of interest] shouldn’t arise in the kind of proposals submitted to the national or regional PHRCs"* (External Reviewer 8 [see Table S1 for external reviewers’ characteristics]). In addition to financial CoIs, four types of non-financial CoIs were identified (Table 3).

**Table 3:** Non-financial conflicts of interests in grant-application peer review: perception, experience and management.

	All participants, n (N=98)	Internal reviewers, n (N=38)	External reviewers, n (N=27)	Applicants, n (N=33)
**Non-financial conflicts of interest (CoI) spontaneously reported**				
Yes	73	28	22	23
No	25	10	5	10
**Type of non-financial CoI experienced or suspected**				
Disciplinary	49	17	17	15
Rivalry or cronyism	28	7	10	11
Geographic	7	4	1	2
Academic	4	0	1	3
**Experience with non-financial CoIs**				
Yes (personal or not)	60	14	22	24
Personal experience	39	11	15	13
**Prevention of non-financial CoIs**				
CoIs viewed as unacceptable	12	4	4	4
CoIs viewed as unavoidable	15	6	5	4
**Management of non-financial CoIs by experts and internal reviewers**				
Always refuses to review	9	5	4	-
Case-by-case decision	8	2	6	-
Accepts to review while directing special attention to impartiality	9	4	5	-

1. Disciplinary conflicts (i.e., competition among specialties or schools of thought) were unanimously listed as the most frequently occurring CoIs, ahead of personal or institutional rivalries, political considerations, and cronyism: *“Conflicts of interest are often disciplinary conflicts. […] That is, each specialty defends itself against other specialties"* (Internal Reviewer 16). The existence of disciplinary CoIs was reported more often by internal reviewers with the national PHRC than by those with the Paris PHRC. “*I noticed that there were some disciplines that supported [their own discipline] a lot. (.) So there were disciplines that strongly supported their topics, [and for example], according to [the president of the board], neurologists have given considerable support to their specialty. (.) So this may seem unfair*" (Internal reviewer 26 [see Table S2 for internal reviewers’ characteristics]).
  During the national PHRC meeting, CP noted a strong reaction of the group against some of the internal reviewers who defended their disciplines too strenuously. The president pointed out to an internal reviewer that not all the projects he/she had reviewed could be perfect.
2. Rivalry or cronyism was mentioned by both the applicants and the reviewers. *“I don’t know whether everyone admits this to you in the same way, but we are all the same, we are much more lenient, well, we are lenient with the people we know"* (External Reviewer 10).
3. Geographic and (4) academic CoIs were particularly likely to occur in competitions among universities or between the Paris region and the rest of France and were less often mentioned by the participants. *“I am concerned when I see that one-quarter of the country [is represented in the reviewing process] with no counteracting factors, because sometimes you can see, I don't know, between Marseille and Lyon or wherever, there can be petty rivalries, unfortunately, it happens*" (Applicant 21 [see Table S3 for applicants’ characteristics]). “*It’s a tremendous problem […] I would say the Paris teaching hospitals are hugely overrepresented [in the national PHRC]. they handle all the funds, for patient care, for research, for teaching, and they have far more professors than the rest of France. which gives them greater operational capacity with respect to their proposals."* (Applicant 28).

### Experience with Non-financial CoIs

Applicants could not formally prove the existence of non-financial CoIs in the grant-application peer review process, but one-third of them (13/38) reported having personal experience with such CoIs. Their suspicion that non-financial CoIs had affected the review process originated occasionally in personal convictions, interpretations, and hearsay and more often in discordances between reviewers’ reports. *“So it is a small world, we know everyone within the disciplines, and it is human, so there are true scientific reviews, and then politics, conflicts of interest, rivalries. jealousies but like any review involving scientific experts, I think we cannot avoid that*" (Applicant 22);*“I don’t have any proof of what I say! I don’t know for sure, I am just guessing"* (Applicant 28); and *“What is a little weird sometimes too, is the gap between two reviewers […], here we will never know."* (Applicant 19). Most applicants were fatalistic about this situation and did not complain despite their suspicions: “*Of course, we always hear about applicants who may be well-connected. because. you know. because the internal reviewers, well the external reviewers who. who are chosen know the applicants or there are conflicts of interest. It is possible, isn’t it? Yes, we hear about that but. what can we do?"* (Applicant 10).

Applicant 29 suspected that an idea was stolen from a previous application he had submitted: *“That can happen, and according to me*… *I made a proposal about a gene and … I saw a database [about that gene] two years later! It could be a coincidence but it is weird! […] They looked for the gene I had proposed in a cohort of patients. […] Now I don’t know for sure, but I have my suspicions."*

### Prevention of Non-financial Cois

While non-financial CoIs were considered either unacceptable or unavoidable by the various stakeholders, opinions about the feasibility of preventing CoIs were more contrasted. Some interviewees were fatalistic (*“It is human […] I think we cannot avoid it"*, Applicant 22), while others were quite satisfied with the current peer review system (*“I don’t have any criticisms to make about the peer review process"*, External Reviewer 22). Moreover, no participants suggested the peer review system should be reconsidered. *“Is there a better system?"* (External Reviewer 16) and *“Who else do you want as reviewers?"* (External Reviewer 23).

Other participants considered that CoIs were too variable in nature to be properly managed: *“It is absolutely unfeasible, because there are fifty different levels of conflicting interests, disciplinary, geographic, personal, you see what I mean…. All kind of networks, in every way, so we can’t manage that… and it goes in all directions, you see what I mean… there are positive conflicts of interests, negative ones […]. For example, something that happens all the time is that people trash others’ proposals in order to open the way for theirs, you see?"* (Internal Reviewer 30).

Interestingly, even when CoIs were suspected, they were not always perceived as important by the internal reviewers. *“I don’t think it matters that much. In practice, it may explain 15% of the variance […], that’s all"* (Internal Reviewer 16). Finally, the internal reviewers felt that, despite rare exceptions, the best applications were selected: *“So, after that, from a pragmatic viewpoint, when all is said and done, we have the feeling that the best proposals are funded"* (Internal Reviewer 12).

In addition, external reviewers had no knowledge of the reporting and management of CoIs during the grant-application review process. More generally, most of them were unaware of how their reviews were considered in the final assessment: *“We don’t have the list of the funded proposals and neither do we get feedback about the reasons for rejections. So, I don’t know in the end, after providing my expertise, how my review was used in the process."* (External Reviewer 16).

### Current Regulation Mechanisms

We found that several mechanisms were used to limit CoIs, although they were not explicitly described in an official policy statement. Grant applicants could list the names of experts they did not want as reviewers of their projects. Experts could, but were not mandated to, refuse to review projects they felt might involve CoIs. Each grant application was reviewed by three (national PHRC) or five (Paris regional PHRC) internal and external reviewers, whose names were masked to the applicants. Moreover, the panel members were chosen from a variety of geographic areas and specialties to ensure that the panel represented the diversity of the grant applications. Grant applications were discussed collectively during the panel meeting, and panel members were free to voice their opinions, although the discussions were influenced by individual factors such as effectiveness in public speaking, scientific expertise, desire to share personal convictions, and willingness to risk expressing disagreement. The panel president ensured that internal reviewer(s) who were involved with an application as investigator were not present when the application was discussed. During the panel meeting, the president played an important role in identifying and managing CoIs, for example by ensuring that internal reviewers did not place excessive emphasis on applications in their own disciplines to the expense of those in other disciplines: *“These proposals are certainly excellent, but all the same, we must consider the others…"* (observed during the national PHRC committee meeting in March 2009).

### Management of Non-financial Conflicts of Interest

Personal experience with non-financial CoIs was reported by 26 reviewers. Based on the interviews of these reviewers, approaches to CoI management were divided into three evenly represented categories.

First, some reviewers routinely refused to review grant applications if they felt they might be biased in favor of or against the applicant: *“I have already refused to review [a proposal] because of conflicts of interest"* (External Reviewer 21) and *“Well, I refused when I received the first letter about [the proposal]… although I was itching to do it…"* (External Reviewer 15). This concern about non-financial CoIs was often based on the existence of personal relationships – positive or negative – with the applicant: *“It happened to me once, no, sorry, twice, to send back a proposal because of conflicts of interest. Twice, because the person who sent me the proposal didn’t realize that I was part of a team that was involved in the research project." (*Internal Reviewer 14).

Second, some reviewers felt that non-financial CoIs were unavoidable and should be managed by conducting the reviews in a strictly impartial manner. They only refused to review applications for which they felt unable to remain impartial: *“I have already reviewed an application for which I had [a CoI]., and I tried to separate myself from any influence of that"* (External Reviewer 12). The problem is recognizing the non-financial CoI: *“Where does it begin? Where does it stop?"* (External Reviewer 11).

The third group of reviewers adopted a case-by-case approach to decide whether or not to review each application according to their subjective understanding of potential non-financial CoIs. For example, two reviewers said that they refused reviews if they were biased against the applicant or project, but not if their bias was positive: *“I am not perfectly honest, because I am too positive, but… in any case, I do not batter a project for reasons that are not purely scientific."* (External Reviewer 14).

### Suggestions for Improvement

Among the numerous suggestions for improving the peer review process (Table 4), masking of applicants was listed most often. *“Obviously, if [my name] had been masked… that would have changed things…"* (Applicant 16) and *“It [applicant name masking] would result in the application being evaluated independently from the research group, its financial resources, whether it received a PHRC grant last year (…) and so the review would be based only on scientific quality. I think it would be better"* (Applicant 19). However, some of the reviewers believed this method would fail in many cases: *“We can guess who it is. We don’t know for sure, but we guess or we believe we know"* (External Reviewer 13). In addition, masking may prevent a valid assessment of the feasibility of the research project: *“I don’t think anonymity matters that much, but it can be harmful, because in the reviewing process, you must know the team (…) if it is blinded, I don’t know who will carry out the project (.) Sometimes a team can write a good proposal but does not have the resources to carry it out! Knowing the clinical research network helps me to say ‘if it is this team or that team, OK, I know it can work’. But if it is blinded, you cannot do that."* (External Reviewer 8).

**Table 4:** Participants’ suggestions to minimize non-financial conflicts of interests (CoIs).

Suggestions	All participants, n (N=98)	Internal reviewers, n (N=38)	External reviewers, n (N=27)	Applicants, n (N=33)
No improvements are possible	7	4	1	2
Masking applicant’s identity	26	7	7	12
Careful selection of independent reviewers	21	6	6	9
International reviewers	18	10	5	3
Possibility for an applicant to challenge a reviewer	6	4	0	2
Open peer review	2	2	0	0
Enhancement of general transparency procedures	17	5	5	7
Interactions with the grant applicant during the reviewing process	12	1	4	7
Public disclosure of conflicts of interest	6	4	1	1
Training of peer reviewers	1	0	1	0

Regarding the overall reviewer selection process, many interviewees voiced major concerns about reviewer selection, especially for specific specialties or research topics: *“Genuine conflicts of interests result from the choice of reviewers who will assess the applications and on their relationships, if any, with the applicant"* (External Reviewer 8). Selecting reviewers from other countries was suggested as a possible solution by some participants: *“We must stop using self-assessment and confining ourselves to the French community. We must avoid the consequences of having French people assess French projects. Other organizations require that the applications be written in English and send them to international reviewers. The [peer review] process is influenced by interpersonal factors. We must steer clear of all relationships that can result in conflicts of interest."* (Internal Reviewer 21). For small disciplines at greater risk for non-financial CoIs, some of the internal reviewers suggested the selection of non-clinical peer reviewers, such as methodologists: *“You will have an external reviewer who is not a specialist, who is not competing with you, and who will give a more objective opinion"* (Internal Reviewer 7).

Another suggestion was to give the applicants the opportunity to challenge the report of the reviewers: *“[We should] have a process for applicants to acknowledge that a reviewer was objective […] or to refuse that a given reviewer assesses their work [as happens with manuscript peer review]"* (Applicant 7).

Improving transparency was also suggested: *“[The proposal] is discussed by the committee, [but] we do not have much transparency about the discussion, the results, how they talk among themselves, and how they rate the applications* …*"* (Applicant 19). *“It is not transparent at all. When someone submits an application, he or she doesn’t know what will happen! Of course, some of us (the reviewers) can tell the applicant [what happened], because we are members of the committee [….] but that is not official policy."* (Internal Reviewer 12). Identifying the reviewers might improve transparency: *“By conviction and for transparency, I believe it would be better if the reviewers were identified"* (External Reviewer 12). However, reviewer anonymity may also have advantages, as explained by the same external reviewer: *“Well, nevertheless, I would prefer it to remain blinded because one can express oneself more easily.[…] And if reviewers were unmasked, well, we might not provide the full extent of our opinions, to keep from offending or hurting someone".* Similarly, according to Applicant 15, *“That’s a good question! Would I like to know my reviewers? No, I don’t think so, it must stay impersonal. No, no, (…) I think it would bring nothing but trouble, particularly in the medical community, where we all … you know… perform favors for one another. I would be embarrassed [to know] that a colleague refused my application."*

Other suggestions were made, such as interactions between applicants and reviewers: *“We should consider, I don’t know, a hearing of the applicant for example, because there can be things that are easier to discuss or to talk about face-to-face"* (External Reviewer 12). *“Peer review by correspondence, or just by talking and asking questions of the applicant who would reply ‘No, you misunderstood, I said that and not that’, [would allow us to] make a more objective review"* (External Reviewer 9).

Disclosure of CoIs, particularly of a non-financial nature, was mentioned by many interviewees as an important transparency procedure that did not appear to be part of PHRC policy: *“I think [disclosure] is left to the morality of reviewers. Well, we chose a system where we trust one another, but we should be able to be more objective, it would not be completely crazy"* (Internal Reviewer 4). *“Conflicts of interest should be disclosed formally, as for articles in high impact factor journals, where we must routinely disclose the presence or the absence of conflicts of interest, and it is something that may be challenged. I mean that if someone complains or something else, or if someone has not disclosed a relevant conflict of interest, it should matter. Here, it should be done routinely. I don’t think it is done at present… But for the reviews, I think conflicts of interest should be disclosed routinely for each applicant, and applicants should be able to challenge the selection of a reviewer in the event of a conflict of interest."* (Internal Reviewer 11).

Finally, one reviewer suggested training of peer reviewers in the identification and management of CoIs and improved uniformity of the peer review process**:** *“Maybe we should have training sessions for reviewers? […] To see what makes a good application. I think it could be really useful to invite peer reviewers to a few training sessions; this might be a good idea? To increase uniformity of reviewers’ work"* (External Reviewer 1).

## Discussion

### Main Findings

Direct observation of panel meetings and interviews with various stakeholders identified non-financial CoIs as a major concern of all parties involved in the process of academic grant-application review. Most of the interviewees spontaneously reported that non-financial CoIs were a major source of bias in the review process and had a greater influence than did financial or industrial CoIs. This high level of concern about non-financial CoIs was in striking contrast to the absence of a formal procedure for non-financial CoI disclosure and management. Although the various stakeholders usually felt that non-financial CoIs were so protean and ubiquitous as to be unavoidable, they also felt that peer review was the best possible evaluation method. The applicants were generally prepared to accept that the review process was not perfect. Among the suggested methods for CoI management two may deserve particular attention, namely, the careful selection of independent reviewers, particularly from other countries and among methodologists; and increased transparency throughout the review process, including a requirement to disclose non-financial CoIs

### Strengths and Weaknesses of the Study in Relation to Other Studies

To our knowledge, this is the first empirical study that used observational scientific methods to investigate non-financial CoIs potentially affecting the grant-application review process.

Few previous studies assessed the opinions of the various stakeholders in the grant-application review process [8], and the originality of our study is its qualitative design. We chose this design to investigate the participants’ attitudes and views without influencing their answers. Indeed, our objective was not to obtain quantitative data or a complete catalog of participants’ views but, instead, to obtain information that might be useful for improving the grant-application review process. All participants volunteered for the interview, and selection biases related to CoIs were prevented by presentation of our study to eligible participants in general terms that did not mention CoIs or peer review biases. However, the non-response rate was high, particularly among external reviewers. Reasons for refusal to participate included lack of time, lack of interest in the grant-application peer review process, or discontent about the lack of credit given to reviewers. Although our objective was not to obtain a representative sample of participants, we selected the external reviewers and applicants by stratified randomization in order to obtain a wide range of perceptions, experiences, and opinions. The reliability of our results was ensured by triangulation (i.e., observational sessions, interviews, and text analysis software) and an analysis by two researchers not involved in grant-application peer review.

Non-financial CoIs are often listed by editors and scientists as important biases [22]–[26]. However, to our knowledge, their presence and nature have rarely been assessed, and most reports focused on industrial or financial CoIs, particularly in peer review by journals [27]–[30]. These disciplinary, academic, or network CoIs may affect the reproducibility [2], [10], [31], transparency, and equity [4], [16], [32] of fund allocation.

Our study was not designed specifically to explore CoIs but addressed instead the overall academic grant-application evaluation process. Nevertheless, as most participants spontaneously mentioned CoIs, this point probably had little impact on information saturation in this qualitative study. Our focus on a single country may limit the external validity of our findings. It could be argued that the French system lacks transparency compared to those used in the UK (Medical Research Council) and US (National Institutes of Health), which are often used as models for European grant-awarding processes. This could be due to the fact that until recently most of the academic research conducted in France relied on permanent structural funds from the government [33], [34], with academic grants being used to fund supplementary studies. Impartiality and honesty on the part of the reviewers are crucial to the academic grant-awarding system used in France. Official policies include only very few rules intended to minimize the impact of CoIs. There are no specific requirements about what to do in the event of non-financial CoIs, and reviewers often decide on their own whether to report these CoIs. However, the PHRC system that was the focus of our study may not be representative of the entire grant-awarding system in France [35], [36].

Efforts to minimize the impact of CoIs focus chiefly on disclosure, as shown in a recent study of several international grant organizations [35]. Of 27 organizations, 22 required written CoI disclosure statements for each grant-application reviewer. However, disclosure statements may also have limitations, as competing interests may be underreported or misreported [37], [38], particularly those of a non-financial nature. Thus, even in systems with a policy of CoI disclosure, the integrity of expertise may depend on the natural probity of the reviewers. In addition, requiring CoI disclosure may be perceived as challenging the integrity of peer reviewers, which may decrease their willingness to volunteer.

### Meaning of the Study Results and Implications for Policymakers

In France, structural public funds available for research have diminished substantially in recent years, leaving a greater role for funding via grants. Consequently, specific measures designed to minimize the impact of non-financial CoIs are required to ensure trust in the grant-application review process and fairness of grant allocation [4], [16]. Table 5 synthetizes various proposals of improvement drawn from the literature and participants’suggestions. Improving transparency is without doubt crucial. Suggestions to improve transparency include mandatory CoI disclosure, transparent review policy procedures, and open peer review:

**Table 5:** Synthesis of proposals for managing non-financial conflicts of interests (CoI) in grant-application peer review.

Proposals drawn from study results and review of the literature	Pros	Cons	Authors’ comments
Masking of applicant’s identity	Requested by the majority of applicants	Useless according to some reviewers May be harmful (because the identity of the applicant provides information on the feasibility and chances of success of the research project) [1] May have no impact on the reviews [1]	Studies of manuscript and grant-application reviews produced conflicting data [1], [54]. Further studies are needed to assess this method in grant-application review
Enhancement of general transparency procedures	Requested by the majority of applicants Might restore applicants’ trust in grant institutions	May be costly and time consuming	Grant institutions should provide more information about their process (via the Internet for example
Public disclosure of Conflicts of Interests (CoI)	Requested by the majority of applicants May restore applicants’ trust in grant review institutions	Difficulty in defining non-financial CoIs [26] May affect reviewers’ privacy	Need to develop requirements for disclosure of non-financial CoIs
Open peer review	Requested by a few applicants	May impact reviewers’ work and objectivity [1]	Further studies are needed to assess this method in grant review
Interactions with the grant applicant during the reviewing process	Requested by some participants Would allow applicants to challenge the review of their project Already used in some grant institutions [55]	Could be costly and time consuming	Need to assess the impact and feasibility of this method in grant review
Elimination of grant review	Bibliometrics to evaluate the applicants ability to successfully conduct useful research	Not requested by the reviewers or applicants Bibliometric methods have several limitations [1] Other methods such as a lottery or random selection are criticized by applicants [1]	Need to assess the impact and the feasibility of these methods.
Improvement of reviewer selection	Selection of international reviewers, for example with no or few CoIs	Difficulty in finding the best reviewer as “there is no such thing as the perfect reviewer"[1] The reviewers felt to be most appropriate may refuse to review the project	Need to recognize the importance of reviewers’ work
Training of reviewers	Requested by a few external reviewers May increase recognition of reviewers’ work	May be costly or time consuming May have no impact on grant review [56]	Need to assess the impact and feasibility of this method in grant review

First, mandatory CoI disclosure would considerably improve the transparency of the review process. CoI disclosure could be required of all internal and external reviewers and of applicants, allowing crosschecking of the information. In practice, most CoI disclosures are related to financial interests, notably with the industry [16], [39]. In our study, financial CoIs rarely exerted a major influence on academic grant-application reviews. Thus, the main challenge may be to ensure the disclosure of non-financial CoIs in addition to financial CoIs. Non-financial CoIs, related to professional collaborations or interpersonal relationships, are more difficult to detect and to describe, and their definition remains debated [26]. They are usually sought via open questions. Whether all professional collaborations and interpersonal relationships should be disclosed, and how the truthfulness of such disclosures could be checked, are important unresolved issues. The International Committee of Medical Journal Editors (ICMJE) has issued requirements for disclosing both financial and non-financial CoIs [40]. Similar requirements might help grant organizations to detect and manage CoIs. Another matter of debate is whether disclosure statements should be available to the public and what the consequences of such availability might be. Public disclosure of CoI would improve transparency and alleviate applicants’ concerns about the impartiality of the reviews. However, public disclosure may also affect the privacy of the reviewers, especially when non-financial CoIs involve personal relationships or families. Studies should be conducted to evaluate the feasibility and impact on review-process perceptions of unrestricted vs. restricted access to standardized CoI disclosure forms [2] and to assess the level of satisfaction of all those involved.

Second, transparency could be improved by giving applicants free access to reports by external and internal reviewers, as well as to the panel meeting discussions. Audio recordings or verbatim transcripts of the meetings may improve the objectivity of the review process and have been assessed in some grant organizations, including the NIH [41]. Meeting minutes or recordings may help to explain discrepancies between the opinions of the experts and the final funding decision. The recordings or transcripts could be prepared in a way that does not reveal the reviewers’ identities.

A third means of improving transparency is open peer review, i.e., the unmasking of reviewers and applicants. This suggestion has generated considerable controversy [42], [43]. Applicants often feel that open peer review would improve the quality of the review process and would lead to greater objectivity of the reviews, whereas reviewers frequently argue that disclosing their identities would adversely affect the objectivity of their work and the independence of their reviews. Open peer review of article manuscripts seems to have no significant impact on peer review quality or rate of manuscript acceptance but increases refusals of potential reviewers to review manuscripts [44]–[46]. Studies should evaluate the impact of open peer review on the grant-application review process.

Fourthly, assignment of applications to reviewers also deserves attention as a means of minimizing non-financial CoIs. Masking of applicant identity is often suggested by applicants as a means of improving the objectivity of the review process. However, the feasibility of a research project may be difficult to assess without knowledge of which principal investigator and research group are involved. In manuscript submission to journals, the masking of applicants’ identities has been shown to reduce biases, particularly geographic biases and academic CoIs [47], without improving peer review quality or manuscript acceptance rates [48], [49]. Further studies should evaluate the impact of masking applicants’ identities in grant-application reviews, especially as feasibility is a key point in the assessment of proposed research projects. Fifthly, appropriate selection of external reviewers is also important in minimizing potential non-financial CoIs. For example, for manuscript reviews, reviewers suggested by authors seem more likely to write favorable reviews [50], [51], and manuscripts by authors sitting on the editorial board may have a higher acceptance rate [30]. We are not aware of studies assessing this issue in the setting of grant-application reviews. In particular, the risk of non-financial CoIs may be particularly high when reviewers are selected for a limited area of research that has only a small number of experts. In these situations, international experts could be asked to review projects. Another problem is the gap between the increasing need for reviews and the decreasing number of reviewers [52]. Failure to recognize the importance of the work done by reviewers may contribute to explain this decrease [35], [53]. Financial incentives or academic recognition have been suggested to remedy this situation [35], [52], [53].

Peer review, although often criticized, is the most widely used method of research grant allocation. Our results indicate the presence of non-financial CoIs in the grant-application peer review process used to allocate academic funds. We believe there is an urgent need to improve transparency, trust, and fairness, particularly by issuing uniform requirements for non-financial CoI disclosure.

There is still a paucity of data on the efficacy and quality of the grant-application peer review process [2]. In the current context of resource scarcity, research should be undertaken to assess whether increasing transparency would improve the efficiency of the peer review process, notably the satisfaction of all those involved. Whether greater transparency would also increase the rate of successful studies is another debate.

## Supporting Information

Table S1 **Characteristics of external reviewers cited in the article.**
(DOC)

Table S2 **Characteristics of internal reviewers cited in the article.**
(DOC)

Table S3 **Characteristics of applicants cited in the article.**
(DOC)
