# Supplementary material for: Non-Financial Conflicts of Interest in Academic Grant Evaluation: A Qualitative Study of Multiple Stakeholders in France
Source: PLoS One. 2012 Apr 9;7(4):e35247. doi: 10.1371/journal.pone.0035247 (PMC3322153; doi:10.1371/journal.pone.0035247)
Supplement: Table S2 — Characteristics of internal reviewers cited in the article. (DOC) [file pone.0035247.s002.doc]

Table S2: Characteristics of internal reviewers cited in the article

| Internal reviewers | Sex | Age (years) | Geographic area | Job title | Specialty |
| --- | --- | --- | --- | --- | --- |
| Internal Reviewer 4 | Male | 50-59 | Paris area | Senior university-hospital physician | Anesthesia |
| Internal Reviewer 8 | Male | 40-49 | Paris area | Senior university -hospital physician | Anesthesia |
| Internal Reviewer 11 | Male | 40-49 | Paris area | Senior university -hospital physician | Biology |
| Internal Reviewer 12 | Male | 40-49 | Paris area | Senior university -hospital physician | Methodology |
| Internal Reviewer 14 | Male | 40-49 | Paris area | Senior university -hospital physician | Medicine |
| Internal Reviewer 16 | Male | 40-49 | Paris area | Senior university-hospital physician | Methodology |
| Internal Reviewer 21 | Male | Unknown | Paris area | Senior university -hospital physician | Medicine |
| Internal Reviewer 26 | Male | Unknown | Other region | Senior university -hospital physician | Medicine |
| Internal Reviewer 30 | Male | 50-59 | Paris area | Senior university -hospital physician | Methodology |
