# Supplementary material for: Non-Financial Conflicts of Interest in Academic Grant Evaluation: A Qualitative Study of Multiple Stakeholders in France
Source: PLoS One. 2012 Apr 9;7(4):e35247. doi: 10.1371/journal.pone.0035247 (PMC3322153; doi:10.1371/journal.pone.0035247)
Supplement: Table S3 — Characteristics of applicants cited in the article. (DOC) [file pone.0035247.s003.doc]

Table S1: Characteristics of external reviewers cited in the article

| External reviewer's identity | Sex | Age (years) | Geographic area | Status | Specialty |
| --- | --- | --- | --- | --- | --- |
| External reviewer 1 | Male | 30-39 | Paris area | Senior university -hospital physician | Psychiatry |
| External reviewer 8 | Male | 50-59 | Paris area | Senior university -hospital physician | Anesthesia |
| External reviewer 9 | Male | 40-49 | Other region | Senior university -hospital physician | Medicine |
| External reviewer 10 | Female | 40-49 | Other region | Senior university -hospital physician | Biology |
| External reviewer 11 | Female | 50-59 | Other region | Senior university -hospital physician | Biology |
| External reviewer 12 | Male | 50-59 | Other region | Physician not working in a university hospital | Biology |
| External reviewer 13 | Female | 50-59 | Paris area | Senior university -hospital physician | Biology |
| External reviewer 14 | Male | 50-59 | Other region | Senior university -hospital physician | Anesthesia |
| External reviewer 15 | Male | 50-59 | Other region | Senior university -hospital physician | Medicine |
| External reviewer 16 | Male | 50-59 | Paris area | Senior university -hospital physician | Medicine |
| External reviewer 21 | Female | 40-49 | Paris area | Senior university -hospital physician | Methodology |
| External reviewer 22 | Male | 60-70 | Other region | Senior university -hospital physician | Medicine |
| External reviewer 23 | Female | 50-59 | Paris area | Senior university -hospital physician | Biology |

Table S1: Characteristics of internal reviewers cited in the article

| Internal reviewers | Sex | Age (years) | Geographic area | Job title | Specialty |
| --- | --- | --- | --- | --- | --- |
| Internal Reviewer 4 | Male | 50-59 | Paris area | Senior university-hospital physician | Anesthesia |
| Internal Reviewer 8 | Male | 40-49 | Paris area | Senior university -hospital physician | Anesthesia |
| Internal Reviewer 11 | Male | 40-49 | Paris area | Senior university -hospital physician | Biology |
| Internal Reviewer 12 | Male | 40-49 | Paris area | Senior university -hospital physician | Methodology |
| Internal Reviewer 14 | Male | 40-49 | Paris area | Senior university -hospital physician | Medicine |
| Internal Reviewer 16 | Male | 40-49 | Paris area | Senior university-hospital physician | Methodology |
| Internal Reviewer 21 | Male | Unknown | Paris area | Senior university -hospital physician | Medicine |
| Internal Reviewer 26 | Male | Unknown | Other region | Senior university -hospital physician | Medicine |
| Internal Reviewer 30 | Male | 50-59 | Paris area | Senior university -hospital physician | Methodology |

Table S1: Characteristics of applicants cited in the article

| Applicant's identity | Sex | Age (years) | Geographic area | Status | Specialty |
| --- | --- | --- | --- | --- | --- |
| Applicant 7 | Male | Unknown | Other region | Senior university -hospital physician | Medicine |
| Applicant 10 | Female | 40-49 | Paris area | Senior university -hospital physician | Anesthesia |
| Applicant 15 | Male | 30-39 | Paris area | Junior university -hospital physician | Anesthesia |
| Applicant 16 | Female | 30-39 | Other region | Other (nurse) | Other |
| Applicant 19 | Female | 40-49 | Other region | Senior university -hospital physician | Medicine |
| Applicant 21 | Female | 50-59 | Paris area | Senior university -hospital physician | Medicine |
| Applicant 22 | Female | 40-49 | Paris area | Physician not working in a university hospital | Medicine |
| Applicant 28 | Male | Unknown | Other region | Senior university -hospital physician | Medicine |
| Applicant 29 | Male | Unknown | Other region | Senior university -hospital physician | Medicine |
